# Good practices for ^68^Ga radiopharmaceutical production

**DOI:** 10.1186/s41181-022-00180-1

**Published:** 2022-10-22

**Authors:** Bryce J. B. Nelson, Jan D. Andersson, Frank Wuest, Sarah Spreckelmeyer

**Affiliations:** 1 Department of Oncology, University of Alberta, 11560 University Avenue, Edmonton, AB T6G 1Z2 Canada; 2 Edmonton Radiopharmaceutical Center, Alberta Health Services, 11560 University Ave, Edmonton, AB T6G 1Z2 Canada; 3 Department of Nuclear Medicine, Charité - Universitätsmedizin Berlin, Corporate Member of Freie Universität Berlin, Humboldt Universität Zu Berlin, and Berlin Institute of Health, Augustenburger Platz 1, 13353 Berlin, Germany

**Keywords:** ^68^Ga-radiolabeling, Gallium-68, Automation, Cyclotron, Radiolabeling, ^68^Ga-tracer, Radiopharmaceuticals

## Abstract

**Background:**

The radiometal gallium-68 (^68^Ga) is increasingly used in diagnostic positron emission tomography (PET), with ^68^Ga-labeled radiopharmaceuticals developed as potential higher-resolution imaging alternatives to traditional ^99m^Tc agents. In precision medicine, PET applications of ^68^Ga are widespread, with ^68^Ga radiolabeled to a variety of radiotracers that evaluate perfusion and organ function, and target specific biomarkers found on tumor lesions such as prostate-specific membrane antigen, somatostatin, fibroblast activation protein, bombesin, and melanocortin.

**Main body:**

These ^68^Ga radiopharmaceuticals include agents such as [^68^Ga]Ga-macroaggregated albumin for myocardial perfusion evaluation, [^68^Ga]Ga-PLED for assessing renal function, [^68^Ga]Ga-*t*-butyl-HBED for assessing liver function, and [^68^Ga]Ga-PSMA for tumor imaging. The short half-life, favourable nuclear decay properties, ease of radiolabeling, and convenient availability through germanium-68 (^68^Ge) generators and cyclotron production routes strongly positions ^68^Ga for continued growth in clinical deployment. This progress motivates the development of a set of common guidelines and standards for the ^68^Ga radiopharmaceutical community, and recommendations for centers interested in establishing ^68^Ga radiopharmaceutical production.

**Conclusion:**

This review outlines important aspects of ^68^Ga radiopharmacy, including ^68^Ga production routes using a ^68^Ge/^68^Ga generator or medical cyclotron, standardized ^68^Ga radiolabeling methods, quality control procedures for clinical ^68^Ga radiopharmaceuticals, and suggested best practices for centers with established or upcoming ^68^Ga radiopharmaceutical production. Finally, an outlook on ^68^Ga radiopharmaceuticals is presented to highlight potential challenges and opportunities facing the community.

## Background

**Fig. 1:**
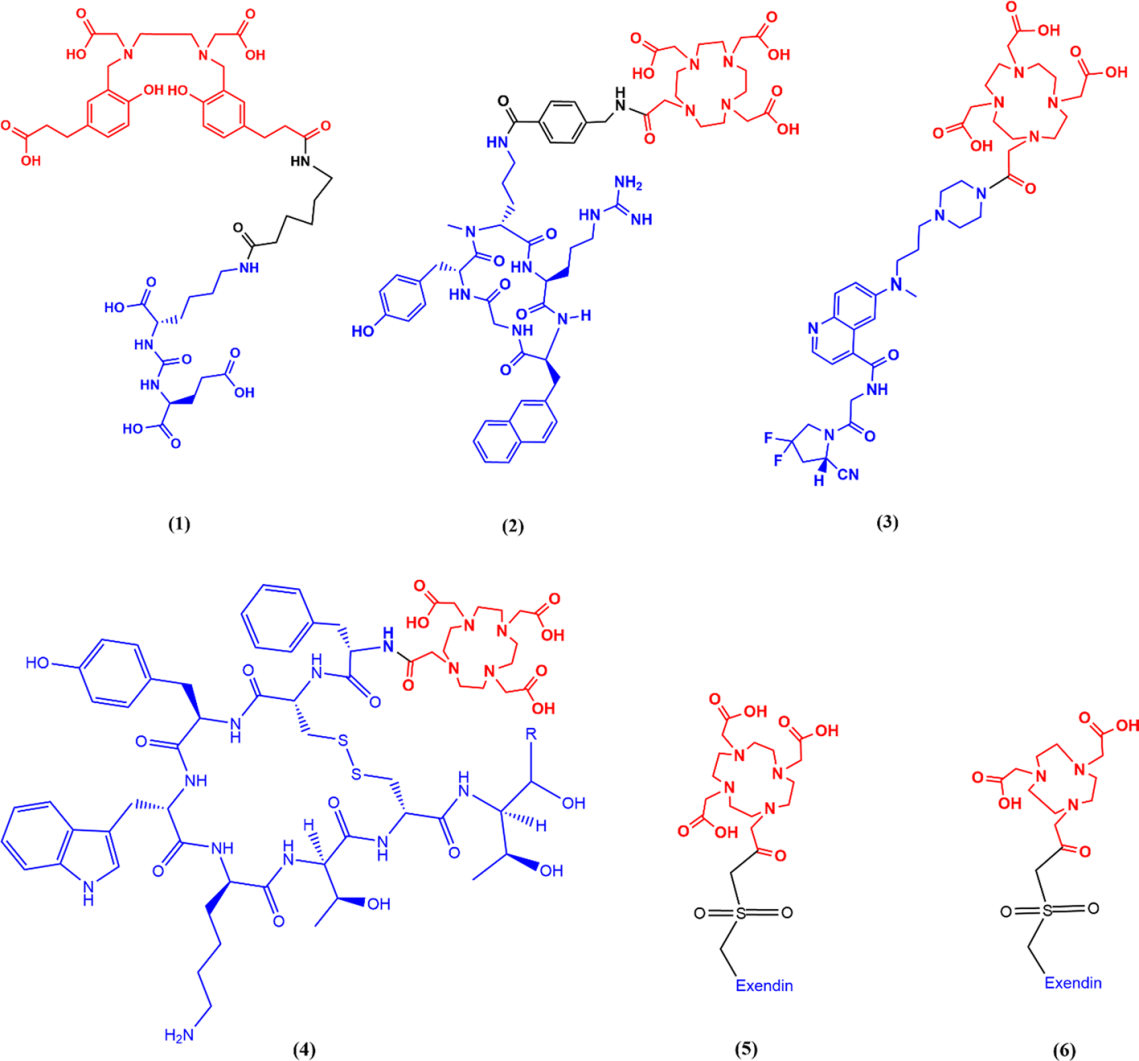
Structures of several ^68^Ga radiopharmaceuticals in clinical use (**1**) PSMA-11 (Fuscaldi et al.^2021^; Hennrich and Eder 2021) (**2**) PentixaFor (Sammartano et al. 2020; Spreckelmeyer et al. 2020) (**3**) FAPI-46 (Spreckelmeyer et al. 2020) (**4**) R = H DOTA-TOC (Bauwens et al. 2010; Decristoforo et al. 2007); R = Carbonyl DOTA-TATE (**5**) Exendin peptide sequence = HGEGTFTSDL SKQ M EEEAVR LFIEWLKNGG PSSGAPPPS C = Exendin-4-Cys40(DOTA) (Velikyan et al. 2017) (**6**) Exendin peptide sequence = HGEGTFTSDL SKQ M EEEAVR LFIEWLKNGG PSSGAPPPS K = Exendin-4-Lys40(NODAGA) (Velikyan et al. 2017; Migliari et al. 2021)

The rise and increasingly widespread clinical use of positron emission tomography (PET) imaging with gallium-68 (^68^Ga) radiopharmaceuticals motivates providing guidance on aspects of ^68^Ga radiopharmaceutical production to aid the community in achieving consistent quality and reliable yields. Radiogallium isotopes have been extensively investigated, starting when gallium was first observed to accumulate at osteogenic activity in the late 1940s (Hayes 1978). Early clinical trials using reactor-produced ^72^Ga (t_1/2_ = 14.1 h) for therapy and diagnostic evaluation of malignant bone lesions were ineffective, with investigation largely stopping by 1952 due to unsatisfactory patient benefits (Hayes 1978). A primary factor contributing to the negative diagnostic results was the poor detection equipment available at the time, while any further attempts exploit ^72^Ga for therapy would have been limited by the high energy and intensity beta particle and gamma ray emissions depositing excess radiation dose in healthy tissue surrounding the tumor sites. Subsequently, accelerator-produced ^67^Ga (t_1/2_ = 3.3 d) was investigated for clinical use, and determined to be an effective tumor and abscess locating agent, with annual usage reaching nearly 250,000 patients by 1977 (Hayes 1978). In 1961, the first ^68^Ga generator system was developed, using decay of germanium-68 (^68^Ge) to provide a continuous supply of ^68^Ga for clinical studies (Gleason 1960). ^68^Ga was viewed as particularly attractive due to its short half-life permitting large activities to be administered for diagnostic imaging, with its rapid decay and clearance preventing excess patient radiation dose. Additionally, ^68^Ga nuclear decay exhibits a high positron branching ratio (88.9%) with minimal co-emitted gamma rays, positioning it favorably compared to other radiometals with respect to dose (https://www.nndc.bnl.gov/nudat2/reCenter.jsp?z=56&n=77). Alongside advances in ^67^Ga, ^68^Ga was initially considered for potential use in PET imaging, however there was insufficient instrumentation at the time to achieve this application. The advent of ^99m^Tc for single photon emission computed tomography (SPECT) imaging and ^18^F for PET imaging delayed the application of ^68^Ga diagnostic imaging owing to widespread ^99m^Tc generator commercial distribution, and the longer half-life of ^18^F compared to ^68^Ga providing ease of production and clinical application. Additionally, early ^68^Ge/^68^Ga generators precluded direct radiolabeling by providing ^68^Ga eluate complexed with EDTA, further slowing the development and utilization of ^68^Ga radiopharmaceuticals (Banerjee and Pomper 2013). With the recent emergence of more advanced PET cameras, and the next generation of GMP-grade commercially available ^68^Ge/^68^Ga generators that reliably provide ^68^Ga in chemically convenient dilute hydrochloric acid, ^68^Ga use for research and clinical application became more widespread. Development and production of many ^68^Ga radiopharmaceuticals ensued for various purposes including myocardial perfusion, renal and liver function, and tumor imaging. Somatostatin (DOTATOC/DOTATATE/DOTANOC) (Bauwens et al. 2010; Decristoforo et al. 2007), prostate-specific membrane antigen (PSMA) (Fuscaldi et al. 2021; Hennrich and Eder 2021), fibroblast activation protein (FAP) (Spreckelmeyer et al. 2020; Loktev et al. 2018), bombesin (Schuhmacher et al. 2005; Richter et al. 2016) and melanocortin 1 (Froidevaux et al. 2004) targeting ^68^Ga radiotracers have been developed (Fig. 1), with their pharmacokinetics often well matched to the short physical half-life of ^68^Ga (Banerjee and Pomper 2013).

With an increasing number of centers using ^68^Ga on a regular basis for research and clinical application, several challenges have been maintaining consistency of reported parameters and providing sufficient process information for preclinical and production data of new ^68^Ga radiopharmaceuticals. This review will present a set of common guidelines and standards would be useful for the ^68^Ga community to report data in a uniform and reliable format. This review aims to outline key aspects of ^68^Ga radiopharmacy, including means of ^68^Ga production and purification via ^68^Ge/^68^Ga generators or medical cyclotrons, standard techniques for radiolabeling compounds with ^68^Ga, and established quality control procedures for clinical grade ^68^Ga radiopharmaceuticals. It also suggests best practices for centers with existing or upcoming ^68^Ga radiopharmaceutical production with respect to preparation of common ^68^Ga tracers, and reporting key production parameters to the community. To conclude, an outlook on the future of ^68^Ga radiopharmaceuticals is presented to highlight some of the upcoming challenges and opportunities presenting the community.

## ^68^Ga production routes: generators and cyclotrons

### ^68^Ga generator production

The most common method for obtaining ^68^Ga is via a ^68^Ge/^68^Ga generator. Generators are convenient for many applications since the 270.93-day half-life of the parent nuclide, germanium-68 (^68^Ge), guarantees an ongoing supply of ^68^Ga sufficient for clinical use for up to a year. ^68^Ga/^68^Ge generators were first developed in the early 1960s, however early generators utilizing liquid–liquid extraction and EDTA eluant to obtain ^68^Ga were not conducive to complex syntheses of ^68^Ga radiopharmaceuticals, and the advent of ^99m^Tc and ^18^F radiopharmaceuticals slowed development of ^68^Ga radiopharmaceuticals in the 1970s (Rösch 2013). Advances in radiochemistry led to availability of new generators providing ^68^Ga^3+^ in hydrochloric acid eluate (Razbash et al. 2005). The eluted ^68^Ga, in the form of [^68^Ga]GaCl_3_, can be used for radiolabeling and has led to significant advances in ^68^Ga chemistry and the development of targeted PET radiopharmaceuticals. Modern commercially available ^68^Ge/^68^Ga generators utilize TiO_2_, SiO_2_, CeO_2_, or SnO_2_ solid phase matrixes to provide [^68^Ga]GaCl_3_ by elution with dilute HCl while the mother ^68^Ge radionuclide remains on the matrix (Table 1). ^68^Ge content is less than 0.001% of ^68^Ga eluate throughout the life of the generator, with the eluate containing minimal metallic impurities (Rösch 2013; Chakravarty et al. 2016; Romero et al. 2020). A recent development is a 4.04 GBq ^68^Ga/^68^Ge generator, capable of producing significantly higher ^68^Ga elution and drug product activities with a longer generator shelf-life compared to previous generators (Waterhouse et al. 2020). The ^68^Ge generator parent radionuclide can be produced via several accelerator-based nuclear transformations, the most common being the ^69^Ga(p,2n)^68^Ge reaction. The cross section for this reaction peaks just under 20 MeV, which is within the range of many medical cyclotrons, however, to achieve reasonable commercial scale yields (> 37 GBq) irradiations of ^69^Ga at 40–100 µA for several days are needed (IAEA PUB1436).

**Table 1:** Commercially available ^68^Ge/^68^Ga generators

Manufacturer	GMP	Matrix	Elution	Size (GBq)
IRE Elit	Yes	TiO_2_	0.1 M HCl	1.85
ITG	Yes	Octadecyl silica	0.05 M HCl	2/4.04 (Waterhouse et al. 2020)
Eckert & Ziegler	Yes	TiO_2_	0.1 M HCl	3.7
iThemba Labs	No	SnO_2_	0.6 M HCl	1.85
Obninsk Cyclotron Co Ltd	No	TiO_2_	0.1 M HCl	3.7
Pars Isotopes	No	nano-SnO_2_	1.0 M HCL	2.59 (Romero et al. 2020)

^68^Ga can also be produced directly on the cyclotron via the ^68^Zn(p,n)^68^Ga nuclear reaction (Tieu et al. 2019; Alnahwi et al. 2020; Lin et al. 2018; Nelson et al. 2020; Thisgaard et al. 2021; Rodnick et al. 2020; Alves et al. 2017; Pandey et al. 2014, 2019; Riga et al. 2018; Jensen and Clark 2011), with various production routes and yields presented in Table 2. Depending on the production technique, cyclotron ^68^Ga yields are typically one to several orders of magnitude greater than currently available ^68^Ga/^68^Ge generators. Significant development has been undertaken in the field of liquid targets for ^68^Ga production. Aqueous solutions of isotopically enriched zinc-68 (^68^Zn) were first subjected to proton bombardment in a regular niobium target mainly used for ^18^F production (Jensen and Clark 2011) and later upgraded to use a niobium foil as a beam degrader, producing 1800 MBq at end of bombardment (EOB) (Riga et al. 2018). Subsequently, a modified target design using an aluminum foil as beam degrader was developed (Pandey et al. 2019) where zinc nitrate in nitric acid was irradiated at 20 µA, producing 9.85 ± 2.09 GBq at EOB. Alternatively, solid targets using electroplated or pressed metal ^68^Zn powder have been used, where ^68^Zn is electroplated or pressed onto metallic target backings. Post-irradiation, the metallic ^68^Zn is dissolved for chemical separation and ^68^Ga purification. An alternative target system combining irradiation and dissolution has recently been developed that aims to address the limitations of solid and liquid targetry.

**Table 2:** Liquid and solid target ^68^Ga cyclotron production routes

Target	Foil	Beam	Yield	References
[^68^Zn]ZnCl_2_	Niobium	15 MeV, 20 µA	1800 MBq EOB	Jensen and Clark (2011)
[^68^Zn]Zn(NO_3_)_2_ (1.7 M) in HNO_3_ (0.2 N)	Aluminum	14 MeV, 20 µA	192.5 ± 11.0 MBq/µA-hr EOB	Pandey et al. (2014)
[^68^Zn]Zn(NO_3_)_2_ (1.7 M) in HNO_3_ (0.2 N)	Niobium	12 MeV, 20 µA	4.3 ± 0.3 GBq	Riga et al. (2018)
1.4 M ^68^Zn(NO_3_)_2_ in 1.2 N HNO_3_	Aluminum	14 MeV, 40 µA, 60 min	9.85 ± 2.09 GBq EOB	Pandey et al. (2019)
100 mg ^68^Zn(NO_3_)_2_	Niobium	14 MeV, 45 µA, 50 min	6 GBq EOB	Alves et al. (2017)
1.0 M ^68^Zn(NO_3_)_2_ in 0.3 N HNO_3_	Niobium/Havar	14.3 MeV, 34 µA, 60 min	4.6 ± 0.4 GBq	Rodnick et al. (2020)
Pressed ^68^Zn	Aluminum	13 MeV, 80 µA, 120 min	194 GBq EOB	Thisgaard et al. (2021)
Pressed ^68^Zn	Aluminum	12.5 MeV, 30 µA, 73 min	37.5 GBq	Nelson et al. (2020)
Electrodeposited ^68^Zn		14.5 MeV, 30 µA, 60 min	60.9 GBq	Lin et al. (2018)
Pressed ^68^Zn		13 MeV, 35 µA, 90 min	145 GBq	Alnahwi et al. (2020)
Electrodeposited ^68^Zn		14.5 MeV, 35 µA, 8.5 min	6.30 GBq	Tieu et al. (2019)

To effectively establish ^68^Ga production, sites should select a liquid or solid target production route based upon their anticipated ^68^Ga demand, available infrastructure, and existing technical expertise. The following sections outline the advantages and disadvantages of liquid and solid ^68^Zn targetry and ^68^Zn/^68^Ga chemical separation techniques.

### ^68^Ga solid and liquid cyclotron targetry

Liquid ^68^Zn target solutions are prepared by dissolving isotopically enriched ^68^Zn metal or ^68^Zn oxide in nitric acid to produce [^68^Zn]Zn(NO_3_)_2_ (Rodnick et al. 2020; Alves et al. 2017; Pandey et al. 2014, 2019; Riga et al. 2018). Alternatively, [^68^Zn]ZnCl_2_ can be employed (Jensen and Clark 2011), however [^68^Zn]Zn(NO_3_)_2_ is preferred, as it was found that irradiating ZnCl_2_ leads to a significant pressure buildup of hydrogen and oxygen resulting from beam-induced radiolysis of the target solution (Pandey et al. 2014). Target assemblies can utilize a combination of helium and water cooling to remove heat, with the target solution and cooling fluids separated by aluminum and niobium foils. Targets are typically irradiated at energies of 12–14 MeV up to 45 µA beam current (Rodnick et al. 2020; Alves et al. 2017; Pandey et al. 2014, 2019; Riga et al. 2018), with ^68^Ga yields dependent on the target pressure and concentration of ^68^Zn solution, yielding up to 9.85 GBq after a 60 min irradiation. While irradiating at higher beam energies increases ^68^Ga yield, it increases production of the ^67^Ga radionuclidic impurity, so irradiating at a lower energy of ~ 12 MeV improves radionuclidic purity through avoiding onset of the ^68^Zn(p,2n)^67^Ga reaction. However trace levels of undesired isotopic impurities (0.1% ^66^Zn and 0.48% ^67^Zn) present in highly enriched ^68^Zn (99.3%) lead to unavoidable production of ^66^Ga and ^67^Ga from the ^66^Zn(p,n)^66^Ga and ^67^Zn(p,n)^67^Ga reactions, respectively (Nelson et al. 2020). To achieve higher beam currents on liquid targets, pressurized target assemblies are required due to cavitation of the target solution. Advantages of liquid targets include ease of solution loading and removal from the target assembly, use of existing cyclotron liquid target infrastructure and similarities to other liquid targetry (^18^F), and shorter ^68^Zn/^68^Ga chemical purification. Limitations of liquid targets include reduced yields resulting from beam energy degradation by the aqueous solution, potential increases in metallic impurities resulting from contact with the target assembly, and heat transfer constraints that limit beam current on-target.

Solid ^68^Zn targets are prepared by either by electroplating or pressing ^68^Zn powder and irradiating at the same beam energies as liquid targets to achieve similar radioisotopic purity. Electroplated targets are produced using platinum, gold, or silver target backings, with the desired cyclotron beam-spot on the target backing immersed in a [^68^Zn]ZnCl_2_ electroplating solution to deposit 40–250 mg ^68^Zn metal on a 7–10 mm target beam-spot (Tieu et al. 2019; Lin et al. 2018; Engle et al. 2012). Targets are then irradiated at currents up to 35 µA beam current at 14.5 MeV, yielding up to 60.9 GBq after a 60 min irradiation (Tieu et al. 2019; Lin et al. 2018). Pressed ^68^Zn targets are manufactured using ^68^Zn metal powder that is compressed within a hardened stainless-steel die into a circular pellet the diameter of the cyclotron beam-spot. ^68^Zn pellets are then sintered onto silver or aluminum target backings and irradiated at beam currents up to 80 µA, yielding up to 194 GBq after a 120 min irradiation (Nelson et al. 2020; Thisgaard et al. 2021; Zeisler et al. 2019). Similar to liquid targets, solid targets use helium and water cooling, with helium cooling provided across the front of the target assembly, while water cooling flows along the target backside. It is important to achieve an even cyclotron beam distribution across the ^68^Zn target material to avoid excessive heat loads concentrated on small areas of the target. For both electroplating and pressed powder targetry, an optimal ^68^Zn pellet thickness can be selected based upon production requirements, with thicker ^68^Zn targets yielding greater ^68^Ga for a given irradiation time at the cost of a greater material expense (Engle et al. 2012). Advantages of ^68^Zn solid targetry include much greater ^68^Ga yields compared to liquid targets, owing to the denser ^68^Zn target material and superior heat transfer to the target backing that enables greater cyclotron beam currents (Nelson et al. 2020). The much higher yields obtained with solid targetry mitigate the short half-life of ^68^Ga, enabling large-scale distribution of ^68^Ga to other PET centers surrounding a cyclotron facility. Limitations of solid targetry include additional dissolution and purification processing steps to separate ^68^Zn from ^68^Ga, the development of additional infrastructure for solid target retrieval and processing, and operator dose associated with processes involving manual retrieval of solid ^68^Zn targets.

To address the limitations of solid and liquid targetry, a combined irradiation-dissolution target system has been developed that combines advantages of liquid and solid targetry while avoiding their major limitations. Irradiation is performed on a remotely actuated multi-position target bar containing seven 20–40 mg solid ^68^Zn metal targets that permits multiple back-to-back irradiations, reducing operator exposure to radioactive dose. The irradiated target material is then dissolved within the target assembly, and the target solution is remotely transferred to a hot cell for ^68^Ga purification via a capillary line. This combined irradiation-dissolution assembly results in a system that combines the ease of use of liquid targets with the high yields of solid targets (https://syniq.hu/product/hybrid-target-system-for-68ga-production).

To select a liquid or solid cyclotron target setup, all of the factors previously discussed should be considered with respect to cyclotron facilities’ infrastructure and technical strengths. Sites may wish to employ liquid ^68^Zn targets if they possess existing liquid target infrastructure and expertise, have a suitable shielded tubing conduit for transferring the irradiated ^68^Zn target solution to a hot cell for processing, and are primarily interested in routine production of smaller activities for use in local PET imaging centers. Alternatively, sites may wish to employ solid ^68^Zn targets if they have existing solid target assemblies and production experience, and aim to produce large activities of ^68^Ga in single batches for many patients and distribute ^68^Ga to more remote PET imaging centers.

### ^68^Zn target processing and ^68^Ga purification

After irradiation, chemical separation must be performed to remove ^68^Zn target material and other metallic impurities that can interfere with subsequent radiolabeling. These separation procedures depend on using specific chemical concentrations and ion exchange column conditions to achieve reliable results, as small deviations in any of these parameters can often have a significant impact on the final ^68^Ga product yield and purity. Liquid and solid target solutions are downloaded to a hot cell via a capillary line containing an automated synthesis unit (such as a TRASIS All-in-One, NEPTIS Mosaic-LC, or GE FASTlab) employing ion exchange chromatography, or solvent extraction. A second ion exchange column is recommended if ^68^Zn target material contains significant levels of metallic impurities that can impact radiolabeling, or if the ^68^Ga elution solution needs to be deacidified. The selection of this secondary column should be based on the chemical purity of the target material and the specific contaminants present in the clinic’s setup. Decay-corrected ^68^Ga yields have ranged from 74 to 96%, depending on the separation process (Nelson et al. 2020; Alves et al. 2017; Pandey et al. 2014).

One liquid target purification involves passing [^68^Zn]Zn(NO_3_)_2_ solution over hydroxamate resin to trap ^68^Ga while eluting ^68^Zn, washing with 0.005 N HNO_3_ to remove residual ^68^Zn, and elution of ^68^Ga using 5.5 N HCl to an AG-1X-8 column. The AG-1X-8 column is subsequently eluted with 2 mL H_2_O to obtain concentrated ^68^GaCl_3_ for radiolabeling (Pandey et al. 2014). Another technique utilizing [^68^Zn]ZnCl_2_ target solution employed a Waters C-18 Sep-Pak, where ^68^Ga sticks to the resin while [^68^Zn]ZnCl_2_ flows through. The Sep-Pak is washed with water, followed by ^68^Ga elution in 0.1 N HCl. The [^68^Zn]ZnCl_2_ is recovered by boiling up the eluate and water washes to the original solution concentration (Jensen and Clark 2011). An additional separation method involved loading [^68^Zn]Zn(NO_3_)_2_ onto a 50W-X8 column, followed by elution with 3 N HCl onto a Biorad 1X8 column, which was then eluted with 0.1 N HCl (Alves et al. 2017).

In contrast, solid ^68^Zn targetry requires an initial dissolution step prior to ^68^Ga separation and purification. Several techniques have employed 10 N HCl to dissolve electroplated or pressed ^68^Zn metal, prior to loading on a BioRad AG50W-X4 cation exchange resin. After washing with 10 N HCl to remove ^68^Zn and other metallic impurities, the ^68^Ga is eluted in 4 N HCl and loaded onto a UTEVA resin column, where the ^68^Ga is eluted in several milliliters of 0.05–0.1 N HCl (Lin et al. 2018; Nelson et al. 2020). ^68^Zn metal can subsequently be recovered from the process solution using an electrolytic cell and manufactured into new targets, and has demonstrated comparable ^68^Ga yields upon irradiation to targets utilizing fresh ^68^Zn (Nelson et al. 2020). An alternative process involves dissolving ^68^Zn in 7 N HNO_3_, followed by adjustment to pH 2 using NH_4_HCO_2_. This solution is passed through a hydroxamate resin, followed by washing with 0.01 N HCl to remove ^68^Zn. ^68^Ga is eluted with 0.75 N HCl, and loaded onto a CUBCX123 resin, washed with 0.01 N HCl, and the final ^68^Ga product is eluted with 5 M NaCl/5.5 N HCl (Alnahwi et al. 2020).

Combined irradiation-dissolution targetry dissolves the ^68^Zn solid target within the target assembly in 7 N HCl, with the dissolution solution remotely transferred via a capillary line to a hot cell where it is loaded onto Zr resin, taking 15–20 min. The resin is washed with 10 N HCl, and eluted with 2 N HCl onto a TK200 resin, where it is washed with 2 N HCl and eluted in 0.05 N HCl. The ^68^Ga[Ga]Cl_3_ product was shown to comply with Ph. Eur. Specifications (https://syniq.hu/product/hybrid-target-system-for-68ga-production). An important consideration when utilizing any of the above methods is the use of concentrated corrosive acids, and particular caution should be taken in order to limit damage to the hot-cells used. Lining the hot cell with protective material, such as a chemically resistant plastic film, is a good option.

The ^68^Zn/^68^Ga chemical separation and purification method should be selected based upon the cyclotron production method, purification time and product requirements. If a liquid target is used to produce ^68^Ga, the resins used in the initial steps of the downstream purification method should be tailored to match the chemistry of the irradiated target solution. Similarly, solid target purification methods should utilize resins conducive to the initial ^68^Zn dissolution step. Owing to the short half-life of ^68^Ga, it is crucial to keep purification time to a minimum, provided it does not excessively sacrifice ^68^Ga product activity yield and radiochemical quality.

## ^68^Ga radiopharmaceutical production techniques

Radiolabeling with ^68^Ga is a single-step synthetic process that can be executed in three ways. ^68^Ga-radiopharmaceuticals can be produced either manually, by (semi)automated processes or by using a cold kit. Each of them will be described and discussed in this section.

### Manual production

The first ^68^Ga-radiopharmaceuticals for human use were prepared manually, since ^68^Ge/^68^Ga generators were not approved by authorities and therefore not available for the broader community. In addition, the demand for ^68^Ga was substantially lower than current use, as existing SPECT nuclear medicine infrastructure was not designed for PET imaging.

**Fig. 2:**
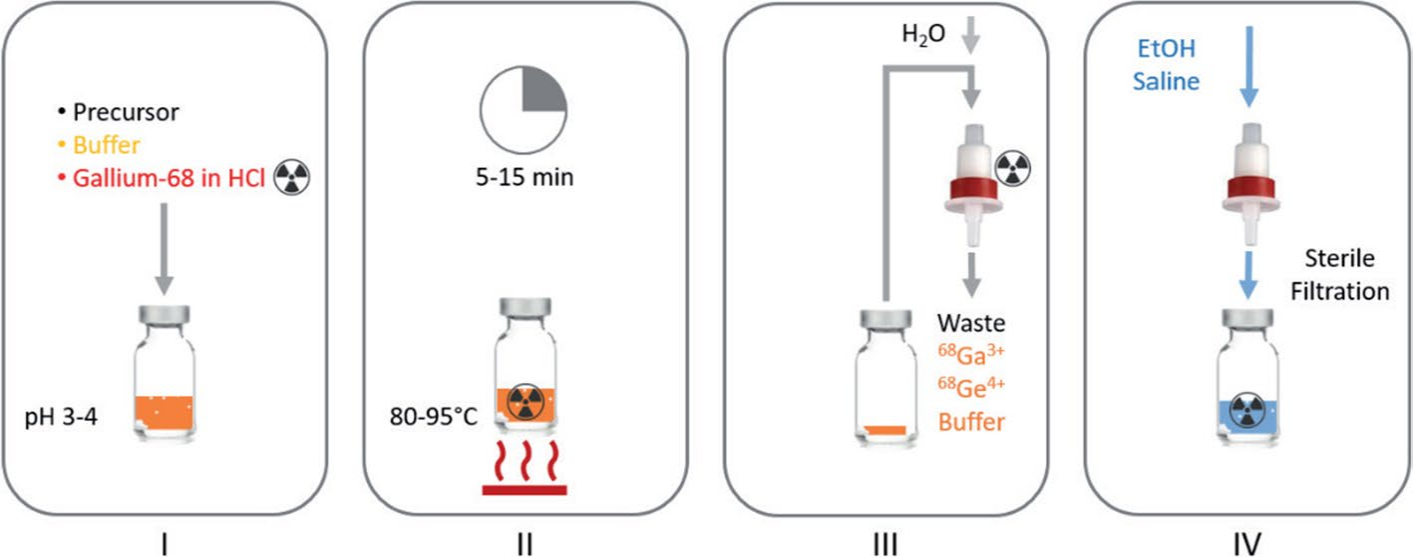
The four main steps of the ^68^Ga-radiolabeling procedure (Meisenheimer et al. 2019)

Figure 2 depicts ^68^Ga-radiolabeling of a DOTA-precursor. First, the reaction mixture is prepared by adding ^68^Ga eluate to a mixture consisting of a suitable buffer, precursor, and additives if necessary. Second, the reaction mixture is incubated for a specific reaction time and temperature to achieve ^68^Ga chelation. Third, the reaction mixture can be purified using a solid phase extraction (SPE) method. The ^68^Ga-radiopharmaceutical is trapped on the column while free ^68^Ga, ^68^Ge impurity, and buffer pass through the column and are discarded. Finally, the product is eluted and passed through a sterile filter as the fourth step (Meisenheimer et al. 2019).

This process can be adapted to different non-DOTA-based precursors, while following the same four general production steps.

A disadvantage of manual production is the manipulation of significant ^68^Ga activities near unshielded hands. The hands of the operator can be only protected from radioactive contamination by gloves, and from dose by extending the distance to the source of radioactivity with tweezers or tongs. Consequently, manual preparation results in high finger dose exposures for the operator (Bauwens et al. 2010). For example, the estimated absorbed body and hand dose for the manual synthesis of [^68^Ga]Ga-DOTA-TATE was 2.0 and 27 µSv, respectively, while compared to an automated synthesis it was 1.3 and 7.9 µSv, respectively (Decker and Turner 2012). Additional disadvantages are inconsistent radiochemical purities and/or radiochemical yields between productions and potentially non-GMP compliant processes, which is not acceptable for translation to a clinical setting.

The key advantage of maintaining full manual control of the radiolabeling process is its usefulness for the early development of radiopharmaceuticals for research purposes. Studies regarding labeling kinetics and radiopharmaceutical stability can be performed, while keeping the hand dose low.

Those labeling studies can be performed with lower radioactivity concentrations to minimize operator dose (< 100 MBq versus 1–2 GBq used for clinical application).

### (Semi)automated production

Due to the increasing clinical demand for ^68^Ga-radiopharmaceuticals and the limitations of manual preparations discussed earlier, there is a need to perform automated.^68^Ga radiolabeling to increase synthesis yields, reliability, and reduce operator dose (Decristoforo 2012). For this purpose, many different synthesis modules are now commercially available, with the majority cassette based. After each synthesis the used cassette is disposed, and a new cassette is installed for subsequent production runs (2019).

**Fig. 3:**
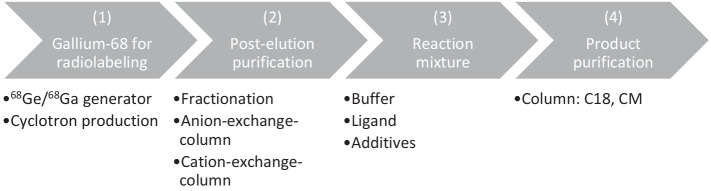
Schematic overview of automated radiolabeling process

Similar to the manual production process, automated production of ^68^Ga-radiopharmaceuticals can be grouped into four main steps, which are outlined in Fig. 3.

First, the ^68^Ga-eluate starting material can be obtained either by a ^68^Ga/^68^Ge generator or cyclotron production as described in Sect. 2. The specifications for the ^68^Ga-eluate are defined in Ph. Eur. (2464) Depending on the source of ^68^Ga, different purification steps are needed before the radionuclide can be used for radiolabeling. In the case of the ^68^Ga/^68^Ge generator, it is important to distinguish if the generator utilizes an organic or inorganic matrix (Velikyan 2015). The eluent for ^68^Ga must be selected based upon the matrix material, and is usually 0.05–2 N HCl, with elution removing the ^68^Ga daughter while the ^68^Ge mother radionuclide remains on the column,. The molarity of the HCl is predefined by the supplier of the generator. A drawback is that the elution volume of the generator ranges between 5 and 10 mL, which is too large for radiolabeling significant activities in the small volumes required for application. Moreover, the eluate can contain metallic impurities resulting from either the matrix material of the column, impurities within the eluent, or the nuclear decay of ^68^Ga (discussed in detail in Sect. 5). In order to purify and concentrate the ^68^Ga-radiolabeling solution to a small volume (~ 200–600 µL), *post-elution processes* are necessary (Decristoforo 2012).

The resulting small volume and higher concentration of ^68^Ga allows for a reduced amount of ligand, and a faster radiolabeling reaction with quantitative incorporation and high tracer specific activity (Velikyan 2015). These post-elution purification processes can be performed in three ways: either by fractionation (Breeman et al. 2005) of the eluate, anion-exchange columns (Meyer et al. 2004), or cation exchange columns (Meisenheimer et al. 2019; Mueller et al. 2016; Zhernosekov et al. 2007).

Fractionation uses the elution fraction with the highest radioactivity concentration without further purification from metal contaminations. Sub-dividing the elution into multiple smaller volumes can capture a significant activity of ^68^Ga in a greatly reduced volume, however substantial activity can be lost in the remaining fractions using this procedure. Additionally, a higher amount of precursor might be needed compared to other purification techniques.

Anion-exchange chromatography absorbs ^68^Ga as an anionic chloro complex [GaCl_4_]^−^. Hydrochloric acid concentrations > 5.5 M form the negatively charged complex that can be adsorbed quantitatively onto strong anion exchange resins. (Meyer et al. 2004) During subsequent elution with H_2_O, ^68^Ga is eluted as ^68^Ga^3+^.^38^

Cation-exchange chromatography uses strong cation exchange columns (SCX) that bind positively charged ions. One of their key advantages is that they can purify the labeling solution from unwanted Ge^4+^, Ti^4+^, Zn^2+^ and Fe^3+^ by eluting these impurities with 97.6% acetone/0.05 N HCl (Zhernosekov et al. 2007). The main drawback of this method is that acetone is an organic impurity that must be avoided in the final formulated product for intravenous injection. Consequently, prior to radiopharmaceutical application, gas chromatography is utilized to verify the amount of acetone is within acceptable limits. To avoid the use of acetone, sodium chloride was found to be a suitable eluent for cation-exchange columns (Velikyan 2015; Zhernosekov et al. 2007).

After purification and concentration of the ^68^Ga-eluate, the solution is introduced into a reaction vessel which contains the *reaction mixture*. The reaction mixture consists of a suitable buffer, the precursor and other additives for radiolabeling. As the requirements for the reaction mixture are universal also for the manual and kit preparation, these are summarized separately in Sect. 4.

After the radiolabeling procedure, the product can be purified if necessary. Most often, C18 cartridges are used to trap the final product. Unreacted ^68^Ga is not trapped on the cartridge and is eluted into the waste fraction. The product and colloidal ^68^Ga stays on the column, while the product is then eluted by an ethanol/water mixture through a sterile filter into the product vial and colloidal ^68^Ga remains on the C18 cartridge. If necessary, HPLC purification can be employed to separate the ^68^Ga radiopharmaceutical from free ^68^Ga and other impurities.

(Semi)automated preparations have advantages compared to manual preparations. They are reliable, reproducible, safe, practical and conform to GMP requirements as they provide digital documentation of the manufacturing process. The transition from a manual to a (semi)automated process is often not straight forward, as changes in parameters used for the manual synthesis may not be applicable for the (semi)automated synthesis. For example, a higher amount of DOTA-TOC was required in an automated process compared to the original manual process (40 µg vs. 30 µg, respectively) (Bauwens et al. 2010). The automation of a radiolabeling process needs to be well-conceived. Instead of expending substantial effort into first optimizing manual radiolabeling, followed by transfer and optimization of the process on a (semi)automated module, it is recommended to immediately start designing the process on the module. Manual radiolabeling typically adds unnecessary time to development since the corresponding automated synthesis can require a re-design and re-development of the entire synthesis process (Pisaneschi and Viola 2021). Additionally, using this development strategy, radiation exposure to personnel that would occur during manual radiolabeling can be avoided.

The (semi) automated synthesis conditions of clinically relevant tracer examples are shown in Table 3. It is important to note that different synthesis modules require different labeling conditions, and when reporting those conditions we encourage use of the template given in Sect. 6 to streamline the adaption of conditions onto different modules.

**Table 3:** Examples of ^68^Ga-radiopharmaceutical synthesis conditions

Precursor	Module	^68^Ga-eluate volume [mL]	^68^Ga-eluate molarity HCl	Buffer	Peptide amount [µg]	Buffer volume [µL]	Pre-concentration	Eluent	Labeling temperature [°C]	Labeling time [min]	Purification	Yield n.d.c. [%] *	Yield d.c. [%]*	Radiochem. Purity HPLC [%]	Total synthesis time [min]
DOTA-NOC (Vis et al. 2015)	ITG	4	0.05	0.25 M Na-acetate	40	1000	none	none	95	10	C18	NA	NA	98	NA
DOTA-TOC (Bauwens et al. 2010)	inhouse	6	0.1	0.5 M NaOAc	40	NA	anionic	0.01 M HCl	90	10	C18	55.1 ± 7.2	NA	99.0 ± 1.2	34.3 ± 3.9
DOTA-TOC (Ocak et al. 2010)	E&Z – Modular Lab	6	0.1	0.5 M HEPES	30	1000	cationic (SCX)	0.02 M HCl + 98% acetone	80 to 100	6	C18	63.9	84.2	> 95	25
PSMA-11 (Fuscaldi et al. 2021)	E&Z – Modular Lab	6	0.1	0.1 M NaOAc	20 (21.12 nmol)	1000	cationic	5 M NaCl/5.5 N HCl	85	5	C18	NA	85.35 ± 5.78	99.06 ± 0.10	25
PSMA-11 (Hennrich and Eder 2021)	Scintomics – GRP	NA	NA	NaOAc	10	NA	cationic	NaCl	100	10	C18	NA	NA	NA	30
Pentixafor (Sammartano et al. 2020)	Scintomics GRP	10	0.1	1.5 M HEPES	20	NA	NA	NaCl 5 M	95	10	C18	57		99.9	33
Pentixafor (Spreckelmeyer et al. 2020)	E&Z – Modular Lab	6	0.1	NaOAc	50	2200	cationic (SCX)	5 M NaCl/5.5 N HCl	95	5	C18	94.7 ± 0.7	80.9 ± 10.0	99.8	NA
Exendin-4 (Velikyan et al. 2017) **	E&Z – Modular Lab	NA	0.1	1 M NaOAc	0.2–20 nmol	NA	cationic (PS-H +)	NaCl/0.05 M HCl	75 (glass); 85 (plastic)	15	tC2	43 ± 2	NA	96.9 ± 0.6	NA
Exendin-4 (Migliari et al. 2021) ***	Scintomics GRP	NA	0.1	1.5 M HEPES	10	3200	Cationic (SCX)	1.5 M NaCl (1.5 mL)	95 (glass)	10	Oasis HLB	NA	23.53 ± 2.4	91.69	NA
DOTA-TATE (Aslani et al. 2014)	E&Z – Modular Lab	8	0.1	0.2 M NaOAc	40	NA	cationic (SCX)	0.02 M HCl + 98% acetone	95	6.67	C18	81.8 ± 0.4, 82.2 ± 0.4, 87.9 ± 0.4		99.5	NA
DOTA-TATE (De Decker and Turner 2012)	IBA molecular – Synthera	NA	0.6	2.5 M NaOAc	20–30 nmol	NA	NA	NA	98	10	NA	56.1 ± 6.2	NA	> 95	NA
DOTA-CP04 (Haskali et al. 2019)	iPHASE Multi Syn	4	0.05	0.5 M NaOAc	30	800	none	none	95	8	Strata X SPE	NA	74.8	92–94	22
FAPI-46 (Spreckelmeyer et al. 2020)	E&Z – Modular Lab	6	0.1	0.4 M NaOAc	50	400	cationic SCX)	5 M NaCl/5.5 N HCl	95	10	CM	96 ± 0.6	95.2 ± 1.4	99.7	NA
	E&Z – Eazy	6	0.1	0.4 M NaOAc	50	400	cationic SCX)	5 M NaCl/5.5 N HCl	98	10	CM	89.7 ± 6.7	NA	NA	NA

*Columns are merged when not stated if n.d.c. or d.c.; NA: not applicable; SCX: Strata X C – Phenomenex ** (DO3A-VS-Cys40-Exendin-4) ***Exendin-4-NODAGA

### Production by using a cold-kit

In analogy to the workhorses in nuclear medicine—^99m^Tc-radiopharmaceuticals—for SPECT imaging, cold kits for ^68^Ga-radiolabeling contain the lyophilized precursor and additional materials such as buffer and stabilizing agents for successful ^68^Ga radiolabeling. Some of the commercially available kits are PSMA-11 (Illumet), DOTA-TATE (Netspot) and DOTA-TOC (Somakit-TOC). The two key components must be chosen wisely – the ligand and the buffer. Excess ligand is likely to have some undesirable effects (solubility, saturation of receptors), whereas too little ligand will result in decreased radiochemical yield (Satpati 2021). An overview of available kits to date are summarized by Satpati (2021). For example, Somakit TOC (2 vial kits) contains 40 µg DOTA-TOC and is radiolabeled with 5 mL ^68^Ga-eluate (HCl 0.1 N solution, maximum activity of 1100 MBq). Another PSMA kit, THP-PSMA by Rotop, contains 40 µg THP-PSMA, sodium bicarbonate, mannitol and phosphate buffer (Derlin et al. 2018). An example of translating a manual labeling process to a kit preparation is outlined in Prince et al., who reported the optimization of [^68^Ga]Ga-DOTA-TATE kit formulation (Prince et al. 2018).

The advantage of cold kits is that they do not need a previous post-processing step of the generator eluate or purification step of the reaction mixture. However, on the contrary, they require manual handling (Meisenheimer et al. 2019). Another advantage is that the production can be performed with limited expertise, although radiopharmacies must become accustomed with using ^68^Ga cold kits since their handling is different than ^99m^Tc-radiopharmaceuticals. Robust preparation is ensured by a long shelf-life, convenient transportation, assured sterility and solubility, procedural reliability and simplicity, and no post-processing of the product (Satpati 2021). Typically, prior to radiolabeling with a kit, 0.5 mL of reaction buffer is added after the elution of the ^68^Ge/^68^Ga generator (Manoharan et al. 2020). The volume of generator eluate must be taken into consideration, as different volumes of acidic ^68^Ga will lead to pH variation that could affect the radiochemical yield.

In summary, sites may select whether to employ (semi)automated production or cold kit techniques based upon their personnel and equipment resources. Sites may pursue semi-automated production if there are sufficient automated synthesis units and automation expertise to implement a production process, large ^68^Ga production activities which preclude manual handling, and if there is no cold kit available for a given tracer. Alternatively, if a fast implementation time is desired with minimal process development work and ^68^Ga production activities are limited, cold kits may be a simpler and effective solution. A typical developmental experiment is to determine the specific activity, which is the lowest possible precursor concentration that achieves complete radiolabeling. For this, we recommend preparing a stock solution of precursor dissolved in water to a concentration of 1 mg/mL. Then, a dilution series of five concentrations ranging from 10^–5^ to 10^–1^ M of the precursor is prepared in a buffer of choice in a small autosampler vial or Eppendorf tube. After the prescribed incubation time, quality control of each sample can be performed via radio-TLC and/or radio-HPLC to determine ^68^Ga radiolabeling incorporation and product integrity.

## Requirements of the reaction mixture

As the post-elution processes use hydrochloric acid, a buffer is required to generate the correct mixture pH for the complexation (Velikyan 2015). The buffer should be nontoxic, have a suitable buffering pH range (e.g. 3.5–5 for DOTATOC), not compete with ^68^Ga^3+^ ions, and preferentially have a weak metal complexing capacity to avoid the formation of colloidal gallium or ^68^Ga(III) precipitate. Moreover, the buffer should be approved for human use. Available buffers include HEPES, acetate, succinate, Tris, glutamate, lactate, oxalate and tartrate. HEPES, succinate, glutamate and oxalate are not approved for human use (Bauwens et al. 2010), although HEPES has optimal labeling characteristics at lower precursor concentrations, maintaining a pH of 3.8 (Velikyan 2015; Sasson et al. 2010).

**Fig. 4:**
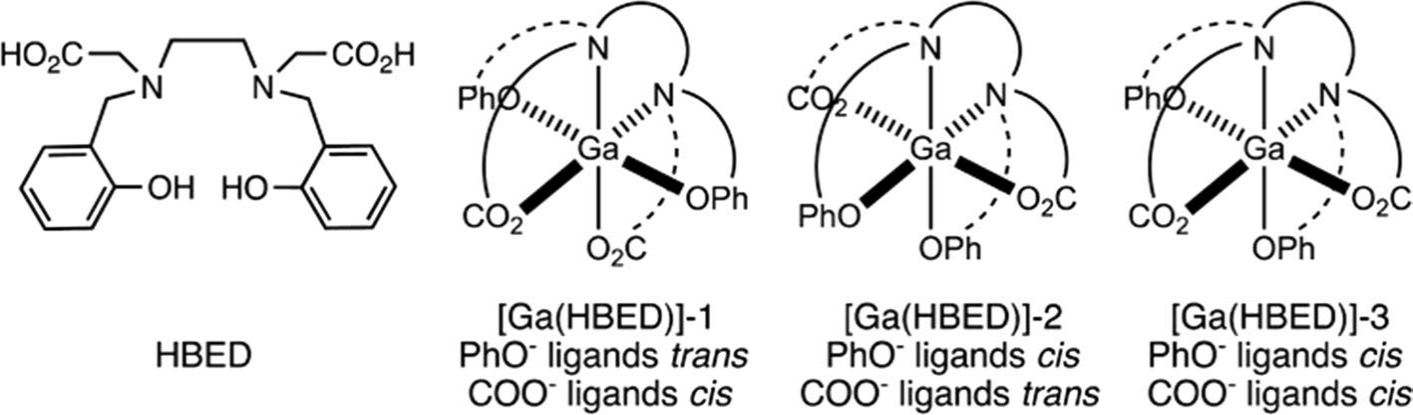
Possible geometric isomers for hexadentate [Ga(HBED)] (Tsionou et al. , 2017)

Regarding the precursor, simple coordination chemistry favors the formation of thermodynamically stable ^68^Ga-complexes with cyclic chelators such as DOTA or NOTA (Morgat et al. 2013), and the acyclic chelator HBED-CC (Price and Orvig 2014; Taliaferro and Martell 1984). The solid state structure of the Ga-DOTA complex was studied extensively by Kubicek et al. (2010). Additionally, Tsiounou et al. (2017) characterized the Ga-HBED complex and proposed the structures seen in Fig. 4. The radiolabeling incorporation and incorporation rate of DOTA-peptides is pH dependent. At pH 1, no incorporation was observed, whereas the [^68^Ga]Ga-DOTA-peptide complex began to form at pH 2.5 and was complete at pH 5 after 5 min at 80 °C (Breeman et al. 2005). The precursor is usually prepared in a 1 mg/mL stock solution and separated into aliquots and frozen at − 20 °C. Some precursors that contain sensitive peptides are freeze-dried into the required volume and prepared fresh on the day of use. For personalized targeting, the precursor contains a chelator (Price and Orvig 2014; Tsionou et al. 2017; Berry et al. 2011), a linker, and a biomolecule targeting vector designed to accumulate with high specificity at a specific target. The precursor may adhere to plastic, glassware and filters when it contains peptides, which may require using higher amounts of peptide based precursors (Breeman et al. 2005). However, low protein binding plasticware can also be utilized to reduce peptide adhesion.

Some radiolabeling procedures require radical scavengers as additives to prevent radiolysis. Due to the high concentration of ^68^Ga, the radiolysis risk of radiosensitive precursors increases. Ascorbic acid, gentisic acid, thiols or ethanol are additives that can be introduced before and/or after complexation (Velikyan 2015).

## Impurities

In this section we will discuss metallic impurities and radionuclidic impurities. Depending on the product requirements and the ^68^Ga radionuclide source, these metallic and radionuclidic impurities can be purified with mini-columns and chemical reduction techniques or permitted if present in sufficiently low concentrations. Other impurities such as chemical impurities or stability related impurities are discussed in Sect. 4.

### Metallic impurities

Metallic impurities play an important role in the quality of ^68^Ga as a starting material (Chakravarty et al. 2016; Cusnir et al. 2019; Ugur et al. 2021; Petrik et al. 2010). In the European Pharmacopoeia (Ph. Eur.) (Ph. Eur. 10.0, 2464) there is a limit of Fe^2+^/Fe^3+^ and Zn^2+^ of 10 µg/GBq, however from a radiolabeling perspective the total amount of metallic impurities that compete with ^68^Ga^3+^ is more important (Cusnir et al. 2019). Apparent molar activity (AMA, GBq/µmol) is a useful parameter as it accounts for all contaminants that compete with the chelator. Metal content can be measured by ICP-MS or ICP-OES, however AMA is also commonly measured by titration curves (Nelson et al. 2020; Svedjehed et al. 2022).

For generators, significant amounts of Zn^2+^ are generated from the decay of ^68^Ga. For a “fresh” 1110 MBq (^68^Ge) generator, the number of stable ^68^Zn atoms generated within one day of an elution is 8.93 × 10^13^ (i.e. 10 ng of Zn^2+^), compared with 4.69 × 10^12^ atoms of ^68^Ga in 800 MBq of eluted ^68^Ga. The amount of stable ^71^Ga generated from ^71^Ge decay may be up to one order of magnitude higher than the amount of stable ^68^Zn generated. In addition, the generator column material can leach residual Ti^4+^ or Fe^3+^. All of these metallic impurities will adversely affect the ^68^Ga labelling yields as well as the apparent molar activity of the labelled product. Thus, dedicated procedures for processing the eluate from the radionuclide generator, including labelling and purification of ^68^Ga radiopharmaceuticals, need to be developed. Several approaches to processing generator derived ^68^Ga^3+^ are described in the literature (Meyer et al. 2004; Breeman et al. 2005; Hofmann et al. 2001; Velikyan et al. 2004).

The trivalent Fe^3+^ is the strongest competitor to ^68^Ga since its chemistry is very similar to that of Ga^3+^, and incomplete removal of Fe^3+^ can lead to incomplete complexation. 1 GBq of ^68^Ga is equivalent to 9.73 × 10^–12^ mol, so even metallic impurities at “low” levels (< ppm) are clearly in excess (Meisenheimer et al. 2019). Many separation techniques have been suggested (see Sect. 3.1.2), and recently Jussing et al. presented a method where ascorbate was used to reduce Fe^3+^ to Fe^2+^, with Ga^3+^ remaining at the higher oxidation state due to its lower reduction potential. The Fe^2+^ can subsequently be separated from Ga^3+^ using a UTEVA resin. Using this updated method, apparent molar activity (AMA) was determined to be in the range of 100 to 200 GBq/µmol for DOTA- based tracers. As a comparison, the clinical batches from generator ^68^Ga had an AMA of 27.5–42.5 GBq/µmol for [^68^Ga]Ga-PentixaFor (Spreckelmeyer et al. 2020) or 13–30 GBq/µmol for [^68^Ga]Ga-FAPI-46 (Spreckelmeyer et al. 2020; Jussing et al. 2021). In the case of potent ligands, the amount that can be administered without induction of pharmacological effects can be small. As an example, for Exendin-4, the maximum amount of the administered peptide was limited to 0.5 µg/kg which would necessitate a high radiopharmaceutical AMA (Velikyan 2015). For cyclotron produced ^68^Ga, special considerations must be taken during separation, however, in general it is recommended to use plastic disposables/contact materials, avoid contact with metal surfaces of working equipment during preparation of reagents, and protect working materials from direct contact with metals. In addition, it is prudent to use trace metal grade chemicals and ultrapure deionized water with a minimum metal content, avoid standard laboratory glassware, and consider coating the fume hood or hot cell with an adhesive plastic lining to avoid corrosion and potential product contamination with Fe (Gallium-68 Cyclotron Production 2019).

### Radionuclidic impurities

The main radionuclidic impurities to be mindful of in ^68^Ga radiopharmaceuticals are ^68^Ge from ^68^Ge/^68^Ga generators, or ^66/67^Ge co-produced during cyclotron production of ^68^Ga. The European Pharmacopoeia states a limit of ^68^Ge of 0.001%. The radionuclidic purity would have to be measured periodically throughout the lifespan of a ^68^Ge/^68^Ga generator to ensure that no ^68^Ge breakthrough is occurring. This is important due to the long half-life of ^68^Ge coupled with an unknown biodistribution in humans. For cyclotron produced ^68^Ga, the ^66/67^Ga impurities are dependent on the target composition, target thickness and irradiation parameters. Irradiating at 12 MeV eliminates the majority of ^67^Ga impurities from the higher energy ^68^Zn(p,2n)^67^Ga reaction, however trace levels of unwanted isotopic impurities (^66^Zn and ^67^Zn) present even in highly enriched ^68^Zn lead to unavoidable production of ^66^Ga and ^67^Ga from the ^66^Zn(p,n)^66^Ga and ^67^Zn(p,n)^67^Ga reactions, respectively (Nelson et al. 2020). Since ^66/67^Ga are radioisotopic impurities of ^68^Ga there is no way of chemically separating them from the ^68^Ga after the irradiation, and depending on the regulations in different jurisdictions, these impurities define or may limit the possible shelf life of the radiopharmaceutical.

In summary, for ^68^Ge/^68^Ga generators it is recommended to diligently test for ^68^Ge breakthrough to ensure radionuclidic purity. For cyclotron ^68^Ga production, it is recommended to optimize the cyclotron beam energy based upon the nuclear reaction cross sections for producing ^66^Ga and ^67^Ga. This beam energy optimization will depend on the proportion of ^66/67^Zn impurities present in isotopically enriched ^68^Zn target material, so calculations should be performed to account for the unique isotopic distribution in different lots of ^68^Zn.

## Summary of ^68^Ga-radiolabeling processes

The critical production step for manual labeling, (semi)automated production, and the use of cold kits is obtaining the right pH in the reaction mixture for quantitative radiolabeling. In theory, this seems an easy task, but the variety of available starting materials, post-elution processes and additives in the reaction mixture makes this challenging. For new radiotracer syntheses, it is recommended to find suitable labeling conditions in manual experiments with low radioactivity and subsequently translate this to an automated process and optimize the conditions on the module, as this is more time consuming and costly compared to the manual process. When optimizing the process, it is important to only change one parameter at a time while keeping the other parameters constant to record the effect of the changed parameter.

Theoretically, the amount of precursor should be as low as possible for two reasons: first, the specific activity will increase when the amount of precursor decreases, reducing potential pharmacological side-effects resulting from the excess of cold precursor. In practice, the tolerated amount of cold precursor should be determined on a case-by case basis. A larger amount of cold precursor can ease requirements of a radiopharmaceutical synthesis if the tracer target does not demand a high specific activity, while some targets such as receptors with low in vivo expression may require a minimal amount of cold precursor to avoid blocking effects. Secondly, the precursor contributes to radiopharmaceutical production cost. By decreasing the amount of precursor, the ^68^Ga radiopharmaceutical cost component for a PET scan will decrease. While the relative cost contribution of the precursor to the entire PET scan cost is typically small and will vary depending on the jurisdiction, this factor should be considered when using more expensive precursors.

When reporting the radiochemical yield of a (semi)automated process, it is sometimes unclear how the yield was calculated. To reduce potential confusion, a standardized method for calculating the absolute non-decay corrected radiochemical yield would be to use activity of the product vial relative to the elution ^68^Ga activity of the generator or cyclotron purification process. The decay corrected yield can then be obtained by a simple calculation. After presenting these yields, any potentially useful information such as distribution of radioactivity within the synthesis process can be discussed.

With regards to troubleshooting and common mistakes, it should be highlighted that utilizing process controls, such as taking probes for quality control assessments every 5 min during the labeling process, are critical to obtaining full control over the labeling mechanism. Using this method, if there is a failed synthesis, the cause can be narrowed down to the pH, labeling temperature, or labeling time. A common mistake is made by exchanging the pre-concentration columns (e.g. from PSH + to SCX) without adjusting the buffer characteristics, which can result in a failed synthesis. Otherwise, the ^68^Ga radiolabeling process is generally straight forward and easy to control.

We would like to encourage authors of radiolabeling procedures to report the following parameters to make it easier for other institutions to adapt the radiolabeling procedure into their clinical routine:

- Module used
- ^68^Ga-eluate volume
- Molarity, volume and name of eluent
- Molarity, volume and name of buffer
- pH of reaction mixture
- Amount of peptide
- Labeling temperature
- Labeling time
- Radiochemical yield n.d.c.
- Radiochemical yield d.c.
- Total synthesis time either from start of generator elution or from introducing the ^68^Ga-eluate received from the cyclotron into the radiolabeling process until the finished sterile filtration of the product
- Apparent molar activity (AMA)

## ^68^Ga radiopharmaceutical quality control

### Specifications

In the Ph. Eur., three ^68^Ga-related monographs can be found—namely “gallium-68 for radiolabeling” (Ph. Eur. 10.0, 2464), “(^68^Ga)Galliumedotreotide solution for injection” (Ph. Eur. 10.0, 2482) and “Gallium (^68^Ga) PSMA-11 injection” (In Ph. Eur. 10.4, 3044). The specifications of ^68^Ga-tracers are summarized in Table 4.

**Table 4:** Specifications of [^68^Ga]Ga-DOTA-TOC and [^68^Ga]Ga-PSMA-11

Test	Specifications [^68^Ga]Ga-DOTA-TOC	Specifications [^68^Ga]Ga-PSMA-11 (In Ph. Eur. 10.4, 3044)	Method
Appearance	Clear, colourless solution	Clear, colourless solution	Visual
pH	4–8	4–8	pH strips
Endotoxins	< 175I.E./V (maximal dose in mL)	< 175I.E./V (maximal dose in mL)	
Radiochemical identity and Purity	> 91% overall purity Colloidal ^68^Ga: < 3%	> 95% overall purity Colloidal ^68^Ga: < 3%	TLC
	Free ^68^GaCl_3_: < 2%	> 95% overall purity	HPLC
Radionuclidic identity	0.511 MeV, 1.077 MeV, sum peak 1.022 MeV	0.511 MeV, 1.077 MeV, sum peak 1.022 MeV	Gamma spectrometry
	62 – 74 min	61 – 75 min	Half-life
Radio nuclidic purity	^68^Ge < 0.001%	^68^Ge < 0.001%	Gamma spectrometry
Residual solvents	Ethanol max. 10% (V/V) or 2.5 g/dose	Ethanol max. 10% (V/V) or 2.5 g/dose	GC
Chemical Purity	Edotreotide, Galliumedotreotide, others < 50 µg/V	PSMA-11, gallium PSMA-11, others < 30 µg/V	HPLC 220 nm detection (DOTA-TOC), 280 nm (PSMA-11)
	HEPES < 200 µg/V	HEPES < 500 µg/V	TLC
Sterility	Sterile	Sterile	Membrane filtration

For intravenous injection of ^68^Ga-tracers, it is straightforward to verify that the injection solution does not contain any visible particles. The method given by the Ph. Eur. is visual inspection. For radiopharmaceuticals this is generally difficult to implement due to the risk of high radiation doses, especially for the eyes. The IAEA recommends to perform the visual inspection through a lead glass shield, use tongs to hold the sample vial against a light beam, and gently shake it to check for the presence of any particulate matters (I. A. E. Agency 2018).

For the radiochemical identity and purity, limits are set for colloidal ^68^Ga and free ^68^Ga which can be detected by radio-HPLC and/or TLC. Another indication for colloidal ^68^Ga is remaining radioactivity on the purification cartridge (e.g. C18) at the end of the synthesis.

The radionuclidic purity of a ^68^Ga generator is tested by the supplier, and often guaranteed for the specified lifetime of the generator. Once a generator is in use, it is recommended to check for radionuclidic impurities on a regular basis by gamma spectroscopy using a high purity germanium detector to determine if there is any ^68^Ge breakthrough. This should be performed at time intervals specified by the generator manufacturer and follow any regulatory requirements in a given jurisdiction. If no testing requirements are given, testing radionucldic purity at least once per month would be a reasonable interval relative to the useful half-life of the generator, and when using a new model of generator, more frequent weekly tests may initially be warranted to precisely track when ^68^Ge breakthrough occurs. When performing these gamma spectroscopy quality control tests, the ^68^Ga eluate sample should be permitted to decay for at least 48 h prior to analysis.

In many syntheses, ethanol is used either to prevent radiolysis and elute the product from the purification cartridge, such as a C18 column. The maximum dose of ethanol must be less than 10% or 2.5 g/dose, as measured by gas chromatography. Since not all radiopharmacies have a gas chromatography system available, it should be tolerated to calculate the theoretical volume of ethanol at the end of synthesis or validate the ethanol content by an external laboratory.

Chemical purity with respect to the ligand amount is important since some ligands may have a pharmacological effect (e.g. Exendin-4 has a dose limiting nausea and vomiting as side-effect; Byetta is injected subcutaneously with a dose of 10 µg) (Velikyan et al. 2017) or toxicological data prevents using higher amounts of ligand.

Chemical purity with respect to the buffer of choice is discussed earlier. For example, although widely used in preclinical models, HEPES is not approved for human use and requires further analysis.

Separating ^68^Ga-labelled compounds from unlabelled precursor is possible with a suitable HPLC method, however often difficult, so typically cold precursor will remain in solution with the ^68^Ga radiopharmaceutical product. The activity (MA) or specific activity (SA) is expressed in SI units as either MBq/µmol to GBq/µmol or MBq/µg to GBq/µg, respectively (Pisaneschi and Viola 2021). When referring to the MA of ^68^Ga-radiopharmaceuticals, the apparent MA is normally used, which is determined by dividing the amount of radioactivity of the product by the amount of precursor used. The amount of precursor is composed of labeled precursor, unlabeled precursor, and precursor complexed with metal impurities (Luurtsema et al. 2021).

An acceptable pH range of 4–8 is given by the Ph. Eur. Roethlisberger et al. (2017) provided an overview of marketed drug products with extreme formulation pH, e.g. Doxycycline with a pH of 2.55 ± 0.75 (1.8–3.3). The authors summarize under which circumstances a deviation from euhydric and isotonic solutions are acceptable (Roethlisberger et al. 2017)

In summary, for preparing ^68^Ga radiopharmaceuticals, specifications on product radionuclidic purity and identity, chemical purity, ligand amount, and acceptable pH should be followed and validated using appropriate analytical techniques.

### QC equipment and methods used

A crucial part of the quality control of ^68^Ga-radiopharmaceuticals is the HPLC method development. A suitable gradient is required to elucidate radionuclidic and UV-sensitive impurities and give sufficient separation of peaks. For that, we recommend starting development with a commonly employed HPLC method utilizing a standard gradient e.g. 100% H_2_O + 0.1% TFA to 100% ACN + 0.1% TFA within 30 min. After sufficient trial-and-error optimization, variants of this gradient tend to achieve adequate separation of the desired ^68^Ga radiopharmaceuticals, free ^68^Ga, and other impurities. After identification of the product peak with the assistance of a reference standard, other peaks in the HPLC chromatogram should be identified and reduced if the peak is due to radiolytic decomposition of the product (as discussed in Sect. 5). After other peaks are identified and a decision is made if the impurities are tolerable or not, the standard gradient can be adjusted. It is important to note that the impurities seen in the standard chromatogram must also stay visible in the adjusted gradient to prevent misleading results. A validation is intended to ensure that the methods are suitable for their intended purpose (Gillings et al. 2020). A chromatographic comparison of the radioactive product peak with its non-radioactive counterpart is suitable as an identification test. Following quality control procedures, it is necessary to measure the product recovery. It is known that certain radiochemical impurities such as free ^68^Ga ions may be retained in the pre-column filters, the tubing or in the injection system (Gillings et al. 2020). This can be evaluated and discussed in addition to an absolute product recovery, determined as the activity of radiolabeled product to the starting ^68^Ga activity obtained from a generator or cyclotron.

For the evaluation of the HEPES concentration in the final product, the Ph. Eur. recommends a TLC system with a HEPES reference standard and visual comparison of the resulting spots. Pfaff et al. presented an improved TLC method as well as an HPLC assay to improve reproducibility and conclusiveness of the results (Pfaff et al. 2018).

These steps outlined to determine ^68^Ga radiopharmaceutical product quality are an important requirement when preparing ^68^Ga radiopharmaceuticals for clinical applications, and all product quality control regulations for a given jurisdiction should be followed. Requirements, quality reviews, and drug approval packages for ^68^Ga radiopharmaceuticals can be found in databases of regulatory bodies such as the European Pharmacopeia and the United States Food and Drug Administration (FDA). Preparation and quality control for existing and new radiopharmaceuticals should be performed in compliance with the regulations of the jurisdiction of drug use (Gillings et al. 2020, 2021, 2022).

### Stability of the product

**Fig. 5:**
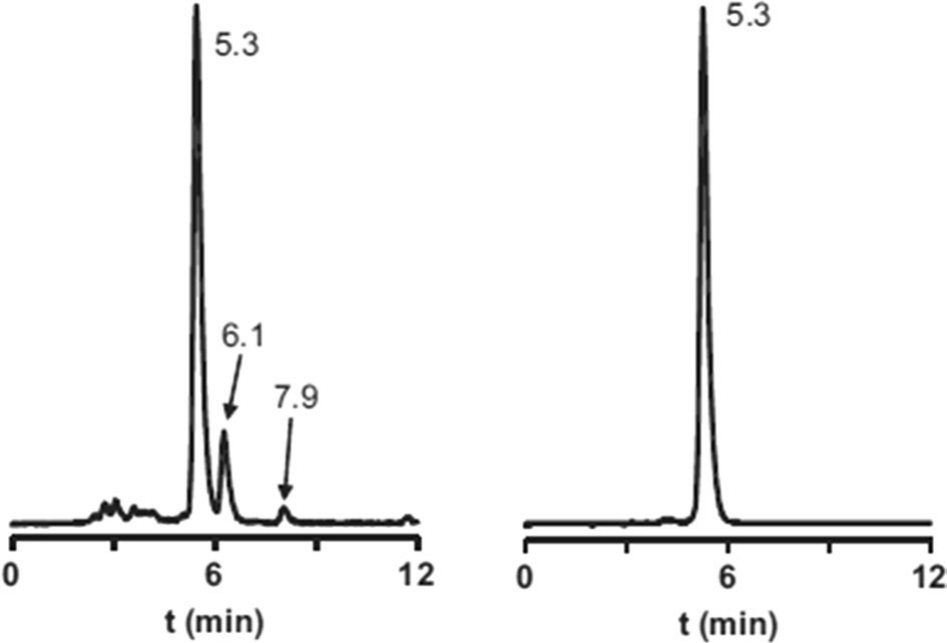
Radio-HPLC chromatogram of 68Ga-DOTA-TATE with the addition of 10% sodium thiosulfate (Left) and with the addition of 10% ethanol (Right) (Mu et al. 2013)

For generator supplied ^68^Ga, the main factor that determines shelf-life of the product is the stability of the radiopharmaceutical from physical and chemical processes. Important factors are radiolytic decomposition as well as oxidation. Stabilizers such as ascorbic acid and/or ethanol can be used to ensure stability over a reasonable shelf life. Shelf stability of the final formulated product must be monitored via radio-HPLC during the validation stage with samples analyzed at different times, at least up to 4 h at room temperature. Stability tests at 4 °C are also recommended (Pisaneschi and Viola 2021). Mu et al. reported the difference for [^68^Ga]Ga-DOTATATE when using different stabilizers and in Fig. 5, HPLC chromatograms from production using 10% sodium thiosulfate or 10% ethanol are presented.

A shelf life of 4 h is usually sufficient for generator supplied ^68^Ga radiopharmaceuticals due to the limited radioactivity that can be obtained. However, since cyclotron produced ^68^Ga offers a significant increase in radiopharmacuetical yields, additional measurements may be warranted if producing larger product activities for use at extended time-points. Unless the immediate area surrounding the radiopharmacy has several PET scanners that can utilize parallel scanning, increasing the shelf life is mandatory for efficient use of the product and shipment to other PET centers. There are several challenges with this, (1) higher starting radioactivity inherently increases the risk for radiolytic decomposition of the radiopharmaceutical, (2) longer shelf life necessitates higher stability of the radiopharmaceutical from oxidation and (3) cyclotron produced ^68^Ga will inherently contain ^66^Ga and ^67^Ga which will increase as a percent of product activity relative to ^68^Ga over the shelf-life duration, since they have significantly longer half-lives. Depending on the exact radionuclidic impurity profile and the applicable regulations, this can limit the shelf life of the radiopharmaceutical in question.

## Conclusions and future outlook

The advent of commercial ^68^Ge/^68^Ga generator suppliers in multiple countries combined with PET centers implementing ^68^Ga cyclotron production has significantly improved the availability of ^68^Ga and enabled widespread use in research and the clinic. Recent high yield ^68^Ga cyclotron production could enable distribution to smaller communities surrounding cyclotron facilities, improving patient accessibility to diagnostic scans, provided that ^66^Ga/^67^Ga impurities in the ^68^Ga product are within regulatory limits. A potential advantage of distributing ^68^Ga activity from a cyclotron facility to smaller surrounding PET centers is lower shielding and processing space requirements associated with producing ^68^Ga radiopharmaceuticals on site from scratch using a ^68^Ge/^68^Ga generator.

Additionally, the widespread availability of ^68^Ga has resulted in its increasing use as a diagnostic radionuclide partner for nuclear medicine theranostics, highlighted by prostate cancer clinical trials using [^68^Ga]Ga-PSMA compounds as a diagnostic imaging agent for [^177^Lu]Lu-PSMA or [^225^Ac]Ac-PSMA therapy (Kratochwil et al. 2016), and [^68^Ga]Ga-DOTATATE used to track [^177^Lu]Lu-DOTATATE or [^225^Ac]Ac-DOTATATE therapy of neuroendocrine tumors (Bal et al. 2021). Since the demand for targeted radionuclide therapies is increasing rapidly, it can be expected that the demand for ^68^Ga theranostic imaging agents will continue to increase.

One downside of ^68^Ga from a ^68^Ge/^68^Ga generator is the low radioactivity per elution, which only enables the scanning of two to three patients. This has resulted in the development of ^18^F tracers for both somatostatin receptors, [^18^F]SiFAlin-TATE (Lindner et al. 2020) and PSMA, [^18^F]DCFPyL (Chen et al. 2011), [^18^F]PSMA-1007 (Cardinale et al. 2017) as alternatives for the equivalent ^68^Ga radiopharmaceuticals. If the purpose of the diagnostic scan is to give an estimation of therapeutic potential for subsequent radionuclide therapy, it helps if the diagnostic agent is structurally related to the therapeutic agent which might be a drawback for generic use of ^18^F-tracers. On the other hand, cyclotron produced ^68^Ga can be supplied in significantly higher radioactivity than generator supplied ^68^Ga but imposes a need for additional infrastructure and a higher regulatory barrier since existing kits only allow generator supplied ^68^Ga to be used as per product monographs.

Due to the development of theranostics, the last ten years has seen an unprecedented development of clinical radiopharmaceuticals, and ^68^Ga-radiopharmaceuticals have been imperative to this progress. With uninterrupted and increasing demand for ^68^Ga radiopharmaceuticals for clinical trials and the FDA approval of multiple ^68^Ga radiopharmaceuticals, compiling ^68^Ga production techniques in a clear and standardized manner will assist PET centers to establish ^68^Ga diagnostic capability, and support the development of new ^68^Ga radiotracers.

## Data Availability

Not applicable.

## Abbreviations

PET: Positron emission tomography
^68^Ga: Gallium-68
^68^Ge: Germanium-68
EDTA: Ethylenediaminetetraacetic acid
DOTATOC: (DOTA(0)-Phe(1)-Tyr(3))octreotid
DOTATATE: DOTA-(Tyr3)-octreotate
DOTANOC: DOTA-1-Nal3-octreotide
PSMA: Prostate-specific membrane antigen
FAP: Fibroblast activation protein
HCl: Hydrochloric acid
GBq: Giga-becquerel
Ref: Reference
EOB: End of bombardement
SPECT: Single photon emission computed tomography
SPE: Solid phase extraction
GMP: Good manufacturing practice
Ph. Eur.: European Pharmacopoeia
SCX: Strong cation exchange columns
HEPES: 4-(2-Hydroxyethyl)-1-piperazineethanesulfonic acid
AMA: Apparent molar activity
ICP–MS: Inductively coupled plasma–mass spectrometry
ICP-OES: Inductively coupled plasma optical emission spectrometry
n.d.c.: Non decay corrected
d.c.: Decay corrected
